# Cerebral Venous Sinus Thrombosis With Bilateral Blindness in Systemic Lupus Erythematosus: A Case Report

**DOI:** 10.1155/crnm/7610185

**Published:** 2026-10-27

**Authors:** Md. Gias Uddin, Mohammad Abul Kalam Azad, Md. Masudul Hassan, Md Moinuddin Sohel, Ismail Hossain, Minhaj Rahim Choudhury

**Affiliations:** ^1^ Department of Rheumatology Bangladesh Medical University Dhaka Bangladesh; ^2^ Department of Medicine Mugda Medical College Dhaka Bangladesh duhs.edu.pk

**Keywords:** bilateral visual loss, cerebral venous sinus thrombosis, neuropsychiatric lupus, optic neuropathy, systemic lupus erythematosus

## Abstract

Cerebral venous sinus thrombosis (CVST) is an uncommon but potentially life‐threatening neurological complication of systemic lupus erythematosus (SLE) that requires prompt recognition and treatment. We report the case of an 18‐year‐old Bangladeshi woman with established SLE who presented with a 10‐day history of severe headache followed by rapidly progressive bilateral proptosis and sudden, painless bilateral visual loss. Neurological examination revealed complete bilateral visual loss, bilateral ptosis, restricted extraocular movements and multiple cranial nerve deficits. She had active SLE with severe thrombocytopaenia and haematological involvement. Brain magnetic resonance imaging and magnetic resonance venography demonstrated extensive thrombosis involving the superior sagittal, bilateral transverse and sigmoid and cavernous sinuses. Antiphospholipid antibody testing, including lupus anticoagulant, anticardiolipin antibodies and Anti‐β2‐glycoprotein I antibodies, was negative. The patient received therapeutic anticoagulation, high‐dose corticosteroids and immunosuppressive therapy with rituximab, followed by haematological and neurological improvement and subsequent recanalisation of the thrombosed dural venous sinuses. However, bilateral visual loss persisted, with optic disc pallor and optic atrophy. Optical coherence tomography, visual evoked potential testing, dedicated orbital imaging and formal neuro‐ophthalmological assessment were not performed; therefore, the precise mechanism of visual loss could not be definitively established. Nevertheless, ischaemic optic neuropathy was considered the most likely mechanism given the clinical presentation and extensive CVST. This case highlights that extensive CVST may occur in active SLE despite negative antiphospholipid antibody testing. It underscores the importance of early neuroimaging, timely anticoagulation and appropriate immunosuppressive therapy to minimise the risk of persistent neurological and visual sequelae.

## 1. Introduction

Systemic lupus erythematosus (SLE) is a chronic multisystem autoimmune disease characterised by autoantibody production and immune complex deposition, resulting in diverse clinical manifestations [1]. Neuropsychiatric involvement is common in SLE and encompasses a broad spectrum of neurological and psychiatric manifestations [2]. Cerebral venous sinus thrombosis (CVST) is an uncommon but potentially life‐threatening neurological complication of SLE [3, 4]. Its pathogenesis is multifactorial and may involve endothelial injury, immune‐mediated inflammation and hypercoagulability. Although antiphospholipid antibodies are an important predisposing factor, CVST can also occur in their absence [5, 6].

Clinically, CVST may present with headache, visual disturbances, cranial nerve palsies, seizures and features of raised intracranial pressure [6, 7]. Early recognition and prompt neuroimaging are essential for diagnosis, while timely anticoagulation and appropriate treatment of the underlying inflammatory disease help limit neurological deterioration and improve clinical outcomes. We report a rare case of established SLE presenting with extensive CVST, persistent bilateral visual loss and multiple cranial nerve palsies despite negative antiphospholipid antibody (aPL) testing.

## 2. Case Presentation

An 18‐year‐old student from Dhaka presented with a 10‐day history of severe headache, predominantly worse in the morning and partially relieved by analgesics. The headache was accompanied by progressive blurring of vision and intermittent vomiting. On the seventh day of illness, she developed bilateral proptosis (Figure 1), followed 3 days later by sudden, painless bilateral visual loss, prompting hospital admission.

**FIGURE 1:**
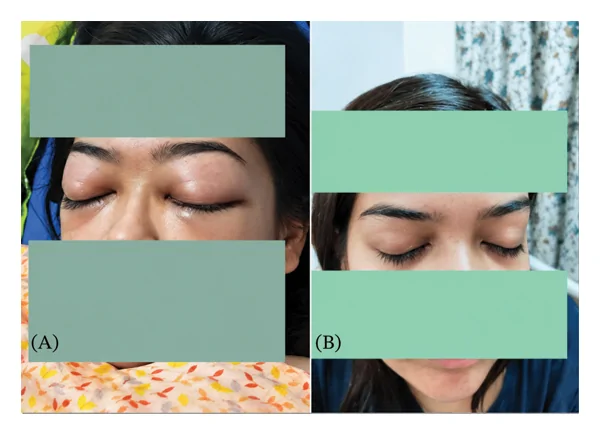
Clinical photographs demonstrating bilateral periorbital oedema before and after treatment. (A) Marked periorbital swelling at hospital admission. (B) Significant improvement in swelling following treatment.

Her past medical history was significant for an unprovoked deep vein thrombosis (DVT) in 2018, at approximately 12 years of age. She was treated with low‐molecular‐weight heparin followed by warfarin for 6 months, after which anticoagulation was discontinued. She remained clinically well thereafter, and an extensive thrombophilia evaluation, including protein C, protein S, Antithrombin III and an aPL panel, was negative.

In 2019, at approximately 13 years of age, she developed polyarthralgia, photosensitivity and exertional dyspnoea. Further evaluation revealed positive antinuclear antibodies, markedly elevated antidouble‐stranded DNA (anti‐dsDNA) antibody levels, severe thrombocytopaenia and pulmonary hypertension, leading to a diagnosis of SLE with immune thrombocytopaenia. Given the age at disease onset, this represented paediatric‐onset SLE.

She was started on hydroxychloroquine, prednisolone and pulmonary vasodilator therapy. Azathioprine (50 mg/day) was introduced as a steroid‐sparing agent but was discontinued after six months because of an inadequate haematological response. Subsequently, mycophenolate mofetil (MMF) was initiated at 2 g/day, resulting in good clinical disease control. Although thrombocytopaenia persisted (20–30 × 10^3^/μL), she remained clinically stable on MMF without major lupus flares or bleeding episodes until the current presentation.

On examination, the patient appeared pale and had bilateral proptosis. Ophthalmological and neurological examination revealed complete bilateral visual loss, bilateral ptosis, restricted extraocular movements and bilaterally dilated, nonreactive pupils. Fundoscopic examination demonstrated bilateral optic disc pallor, consistent with optic atrophy (Figure 2). Optical coherence tomography (OCT), visual evoked potential testing, dedicated orbital imaging and formal neuro‐ophthalmological assessment were not performed. Therefore, the precise mechanism of the visual loss could not be definitively established.

**FIGURE 2:**
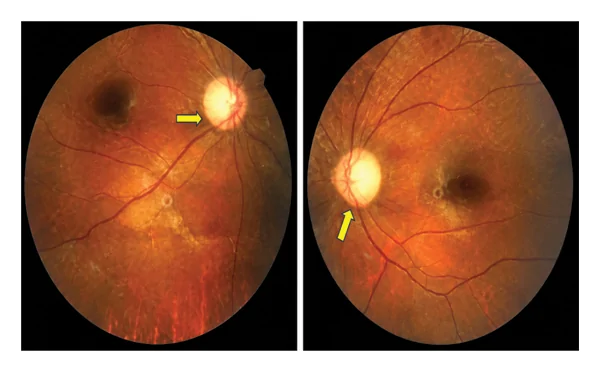
Fundus photographs of both eyes show bilateral optic disc pallor with well‐defined disc margins and attenuated retinal vessels. Arrows indicate areas of optic disc pallor, consistent with bilateral optic atrophy.

Laboratory and ancillary investigations (Table 1) performed at initial presentation showed anaemia, leukopenia and thrombocytopaenia, along with elevated inflammatory markers and an elevated D‐dimer level. Immunological investigations showed a positive antinuclear antibody (ANA) with a homogeneous pattern, markedly elevated anti‐dsDNA antibody levels and reduced Complement C3 and C4 levels, supporting active SLE. APL testing, including lupus anticoagulant, Anti‐*β*2‐glycoprotein I antibody and anticardiolipin antibody, was negative. Urinalysis and renal function tests showed no evidence of lupus nephritis, while hepatic biochemical parameters were within the reference ranges. Serum albumin was mildly reduced. Transthoracic echocardiography demonstrated an estimated pulmonary artery systolic pressure (PASP) of 51 mmHg, suggestive of pulmonary hypertension. Electrocardiography and abdominal ultrasonography were unremarkable.

**TABLE 1:** Summary of laboratory and ancillary investigations at presentation.

Investigation	Patient value	Reference range
Haemoglobin (Hb)	9.5^∗^	12.0–15.5 g/dL
White blood cell count (WBC)	2.5^∗^	4.0–11.0 × 10^3^/μL
Platelet count	45^∗^	150–450 × 10^3^/μL
Erythrocyte sedimentation rate (ESR)	40^∗^	0–20 mm/h
C‐Reactive protein (CRP)	15^∗^	< 6 mg/L
Urine routine examination	No proteinuria, haematuria, pyuria, or urinary casts	Normal
Antinuclear antibody (ANA)	Positive; (homogeneous pattern by indirect immunofluorescence)	Negative
Antidouble‐stranded DNA (anti‐dsDNA)	420^∗^	< 30 IU/mL
Complement C3	0.34^∗^	0.90–1.80 g/L
Complement C4	0.06^∗^	0.10–0.40 g/L
D‐dimer, μg/mL	2.74^∗^	< 0.5 μg/mL
Direct and indirect antiglobulin tests	Negative	Negative
Aspartate aminotransferase (AST)	35	< 37 U/L
Alanine aminotransferase (ALT)	24	10–40 U/L
Lupus anticoagulant	Negative	Negative
Anti‐β2‐glycoprotein I antibody	Negative	Negative
Anticardiolipin antibody	Negative	Negative
Serum creatinine	83	61.9–114.9 μmol/L
Serum albumin	33^†^ ^∗^	35–50 g/L
Sodium (Na_+_)	142	136–145 mEq/L
Potassium (K_+_)	3.6	3.5–5.1 mEq/L
Thyroid‐stimulating hormone (TSH)	4.85	0.25–5.00 μIU/mL
Electrocardiography (ECG)	Normal sinus rhythm; no significant abnormalities	—
Transthoracic echocardiography	Pulmonary artery systolic pressure (PASP): 51^∗^	≤ 35 mmHg
Abdominal ultrasonography	Unremarkable	—

*Note:* Abnormal value. ALT, alanine aminotransferase; ANA, antinuclear antibody; anti‐β2‐GPI, Anti‐β2‐glycoprotein I antibody; anti‐dsDNA, antidouble‐stranded DNA antibody; AST, aspartate aminotransferase; ECG, electrocardiography.

Abbreviations: ALT, alanine aminotransferase; ANA, antinuclear antibody; anti‐β2‐GPI, anti‐β2‐glycoprotein I antibody; anti‐dsDNA, anti‐double‐stranded DNA antibody; AST, aspartate aminotransferase; CRP, C‐reactive protein; ECG, electrocardiography; ESR, erythrocyte sedimentation rate; PASP, pulmonary artery systolic pressure; TSH, thyroid‐stimulating hormone; WBC, white blood cell count.

^∗^Abnormal value.

^†^Serum albumin was originally reported as 3.3 g/dL and is presented here as 33 g/L after unit conversion.

Contrast‐enhanced brain magnetic resonance imaging (MRI) demonstrated the characteristic empty delta sign, with a central filling defect within the superior sagittal sinus surrounded by peripheral contrast enhancement, consistent with CVST. Bilateral mastoid air‐cell opacification was also noted (Figure 3).

**FIGURE 3:**
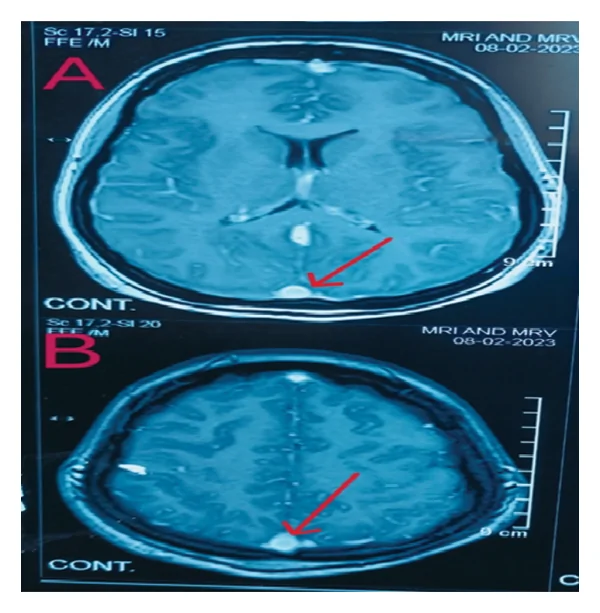
Contrast‐enhanced magnetic resonance imaging (MRI) demonstrating the empty delta sign of superior sagittal sinus thrombosis. Axial (A) and coronal (B) postcontrast T1‐weighted images demonstrate peripheral enhancement surrounding a central nonenhancing filling defect within the superior sagittal sinus (red arrows), producing the characteristic triangular appearance of the empty delta sign. The finding represents contrast‐enhanced blood flowing around an intraluminal thrombus and is consistent with cerebral venous sinus thrombosis.

Magnetic resonance venography (MRV) confirmed extensive thrombosis involving the superior sagittal, bilateral transverse and sigmoid and cavernous sinuses (Figure 4).

**FIGURE 4:**
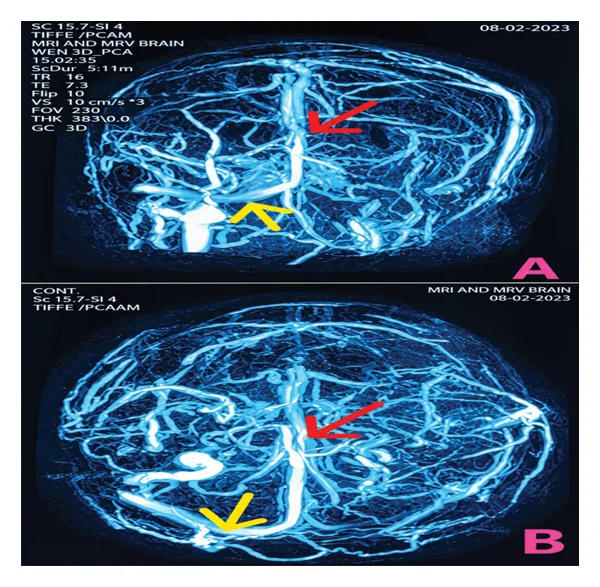
Initial magnetic resonance venography demonstrating extensive cerebral venous sinus thrombosis. The examination shows thrombosis involving the superior sagittal sinus, bilateral transverse and sigmoid sinuses and cavernous sinuses, consistent with extensive dural venous sinus thrombosis.

Bilateral mastoid air‐cell opacification was considered an incidental radiological finding because the patient had no fever, otological symptoms or other clinical or laboratory evidence of active mastoid infection.

The differential diagnosis included acute ischaemic stroke, intracerebral haemorrhage, optic neuritis, idiopathic intracranial hypertension, cavernous sinus syndrome, orbital cellulitis, meningoencephalitis and septic CVST. Septic CVST and orbital infection were considered unlikely because of the absence of fever, otological symptoms and other clinical or laboratory evidence of active infection. The characteristic empty delta sign on contrast‐enhanced MRI, together with MRV‐confirmed thrombosis involving multiple dural venous sinuses, established the diagnosis of extensive CVST.

Based on the clinical, laboratory and neuroimaging findings, we diagnosed extensive CVST in the setting of active SLE. Given the persistently negative aPL testing, we did not support antiphospholipid syndrome (APS). The thrombotic event was considered most likely related to the prothrombotic state associated with active SLE, although other thrombotic risk factors could not be completely excluded.

Despite severe thrombocytopaenia (platelet count, 45 × 10^3^/μL), we initiated therapeutic‐dose low‐molecular‐weight heparin (1 mg/kg twice daily) because of the extensive thrombosis and the high risk of thrombus propagation and neurological deterioration. No intracranial or other major haemorrhagic complications occurred during treatment. High‐dose intravenous methylprednisolone was administered for three consecutive days, followed by oral prednisolone (0.5 mg/kg/day). Rituximab was administered as two 1‐g infusions given 2 weeks apart for refractory immune thrombocytopaenia and active SLE.

Rituximab was selected because of active SLE with refractory immune thrombocytopaenia despite previous treatment with corticosteroids, hydroxychloroquine, azathioprine and MMF. Cyclophosphamide was considered but not selected because there was no evidence of lupus nephritis or other major inflammatory organ involvement requiring cyclophosphamide‐based induction therapy.

After clinical stabilisation and improvement in the platelet count, anticoagulation was transitioned to oral warfarin with a target international normalised ratio (INR) of 2.0–3.0.

Given her history of an unprovoked DVT and subsequent extensive CVST, long‐term anticoagulation was recommended because of her recurrent venous thrombotic events, and she remains on warfarin. Maintenance therapy consisted of hydroxychloroquine (300 mg/day), MMF (2 g/day), ambrisentan (10 mg/day) and warfarin.

During follow‐up, the patient demonstrated marked improvement in headache, bilateral proptosis, ophthalmoplegia and cranial nerve deficits. Platelet counts normalised, and follow‐up MRV demonstrated progressive recanalisation of the previously thrombosed dural venous sinuses (Figure 5).

**FIGURE 5:**
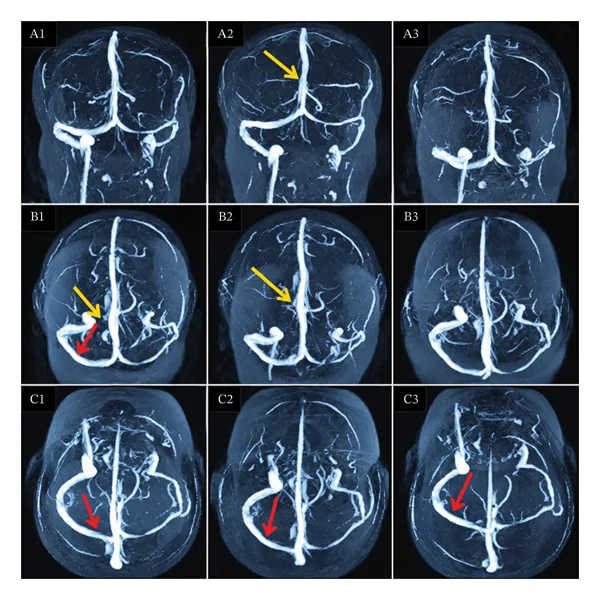
Serial magnetic resonance venography demonstrating progressive recanalisation of the previously thrombosed cerebral dural venous sinuses. (A1–A3) Initial coronal three‐dimensional phase‐contrast MRV demonstrating reduced or absent flow‐related signal within the affected dural venous sinuses, consistent with cerebral venous sinus thrombosis. The yellow arrow in A2 indicates the superior sagittal sinus. (B1–B3) Interim follow‐up MRV demonstrating partial restoration of flow‐related signal within the previously affected dural venous sinuses. The yellow arrows in B1 and B2 indicate the superior sagittal sinus and adjacent posterior dural venous drainage region, while the red arrow in B1 indicates the transverse sinus. (C1–C3) Subsequent follow‐up MRV demonstrates marked restoration of flow‐related signal within the previously affected transverse sinus (red arrows), with preserved flow through the superior sagittal sinus and posterior dural venous drainage pathways. Overall, the serial MRV findings demonstrate progressive recanalisation of the previously thrombosed dural venous sinuses following treatment. MRV figure as a 4096 × 4096 px, 300‐dpi.

However, bilateral visual loss persisted, with established bilateral optic atrophy. Without OCT, visual evoked potential testing, dedicated orbital imaging or formal neuro‐ophthalmological assessment, the precise mechanism of visual loss could not be definitively established. Given the clinical presentation and extensive CVST, particularly cavernous sinus involvement, ischaemic optic neuropathy was considered the most likely mechanism underlying the persistent visual loss. At the most recent follow‐up, the patient remained clinically stable on long‐term warfarin anticoagulation (target INR, 2.0–3.0), together with hydroxychloroquine, MMF and ambrisentan, without recurrent thrombotic events, bleeding complications or lupus flares (Figure 6).

**FIGURE 6:**
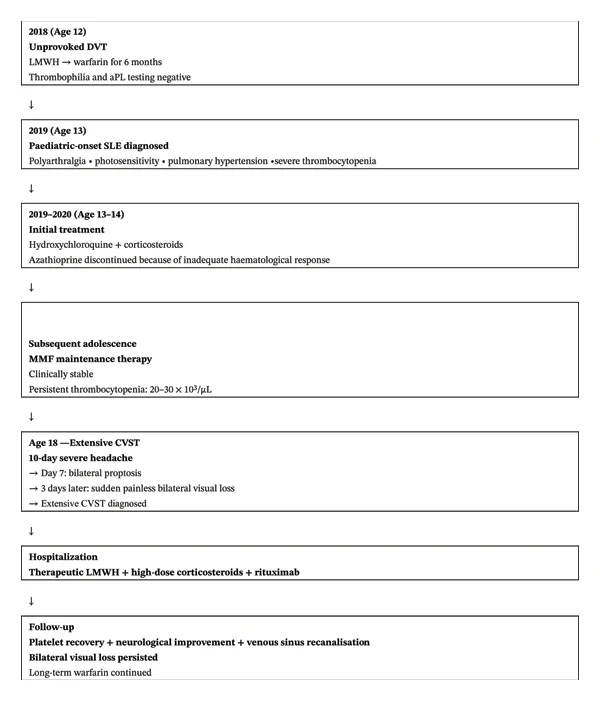
Clinical timeline of paediatric‐onset systemic lupus erythematosus (SLE), recurrent venous thrombosis and extensive cerebral venous sinus thrombosis (CVST). The timeline summarises the patient’s major clinical events, treatments and outcomes from the initial unprovoked deep‐vein thrombosis (DVT) in 2018 and diagnosis of paediatric‐onset SLE in 2019 through the development of extensive CVST at age 18. It highlights the progression from severe headache to bilateral proptosis and sudden, painless bilateral visual loss, followed by treatment with therapeutic low‐molecular‐weight heparin, high‐dose corticosteroids and rituximab. Follow‐up demonstrated platelet recovery, neurological improvement and venous sinus recanalisation; however, bilateral visual loss persisted, and long‐term warfarin therapy was continued.

## 3. Discussion

CVST is a rare but potentially life‐threatening neurological complication of SLE, reported in approximately 0.36% of hospitalised patients with SLE [6]. CVST may present with headache, visual disturbances, seizures and focal neurological deficits, particularly in patients with extensive venous sinus involvement [6, 7]. In our patient, headache progressed to bilateral proptosis, sudden painless bilateral visual loss and multiple cranial nerve palsies involving the oculomotor, trochlear and abducens nerves, in association with extensive thrombosis involving the cavernous and superior sagittal sinuses [6, 8].

An important feature of this case is the paediatric onset of SLE. The patient experienced an unprovoked DVT in 2018 and subsequently developed polyarthralgia, photosensitivity, severe thrombocytopaenia and pulmonary hypertension, leading to an SLE diagnosis during adolescence in 2019. Thus, she had established paediatric‐onset SLE and had been receiving treatment for several years before developing CVST. Paediatric‐onset SLE is often associated with substantial disease burden and may follow a more aggressive clinical course than adult‐onset disease [2, 5]. In this patient, the previous DVT, persistent thrombocytopaenia, active SLE and pulmonary hypertension may have contributed to her overall thrombotic risk, although a direct causal relationship cannot be established.

The differential diagnosis included acute ischaemic stroke, intracerebral haemorrhage, optic neuritis, idiopathic intracranial hypertension, cavernous sinus syndrome, orbital cellulitis, meningoencephalitis and septic CVST. The absence of fever, otological symptoms and other clinical or laboratory evidence of active infection made septic CVST and orbital infection less likely. MRI and MRV confirmed extensive thrombosis involving multiple dural venous sinuses. Given active SLE, severe immune thrombocytopaenia and negative aPL testing, we considered the event most consistent with CVST in the setting of active SLE, with APS less likely [6, 8, 9].

Although CVST in patients with SLE may be associated with APS, our patient tested negative for lupus anticoagulant, anticardiolipin antibodies and Anti‐β2‐glycoprotein I antibodies. In the absence of detectable antiphospholipid antibodies, the thrombotic event was considered less likely to be related to APS and may instead have reflected lupus‐associated inflammation, endothelial dysfunction and a prothrombotic state [8, 9]. This case emphasises that negative aPL testing does not exclude thrombotic complications in patients with active SLE [6, 8].

Persistent severe immune thrombocytopaenia further complicated management. Although severe thrombocytopaenia increases bleeding risk and complicates anticoagulation, thrombotic complications may nevertheless occur in patients with active SLE [6, 8]. Given the extensive CVST and risk of thrombus propagation, we initiated therapeutic‐dose LMWH despite thrombocytopaenia, with careful monitoring and no haemorrhagic complications. After platelet recovery, anticoagulation was transitioned to warfarin with a target INR of 2.0–3.0. This approach illustrates the importance of carefully balancing thrombotic and bleeding risks when therapeutic anticoagulation is required.

Neuroimaging was crucial for diagnosis. Contrast‐enhanced MRI demonstrated findings consistent with dural venous sinus thrombosis, while MRV confirmed extensive thrombosis involving multiple dural venous sinuses [6]. Prompt anticoagulation, high‐dose corticosteroids and immunosuppressive therapy were initiated. Rituximab was subsequently administered for active SLE with refractory immune thrombocytopaenia, after which marked haematological improvement was observed [10]. However, bilateral visual loss persisted. The precise mechanism of visual loss could not be definitively established because OCT, visual evoked potential testing, dedicated orbital imaging and formal neuro‐ophthalmological assessment were not performed. Nevertheless, given the clinical presentation, extensive CVST, cavernous sinus involvement and subsequent optic disc pallor and optic atrophy, ischaemic optic neuropathy was considered the most likely mechanism. Visual impairment may also occur with raised intracranial pressure and optic nerve injury, although evidence from idiopathic intracranial hypertension cannot be directly extrapolated to CVST [11].

Although mastoiditis is a recognised cause of septic CVST, bilateral mastoid air‐cell opacification was considered less likely to represent active mastoiditis because of the absence of fever, otological symptoms and other clinical or laboratory evidence of active infection. The overall clinical context therefore supported a noninfectious mechanism occurring in the setting of active SLE [6, 8].

Transthoracic echocardiography revealed pulmonary hypertension (PASP, 51 mmHg). Pulmonary hypertension is a recognised cardiopulmonary manifestation of connective tissue diseases, including SLE [12]. Although a direct relationship between pulmonary hypertension and CVST cannot be established in this patient, its presence is consistent with the multisystem involvement of her underlying SLE. Compared to previously reported cases, this case has several distinctive features. Extensive dural venous sinus thrombosis occurred despite negative aPL testing, with severe bilateral visual loss, proptosis and multiple cranial nerve palsies. Profound immune thrombocytopaenia also complicated anticoagulation decisions. Although rituximab was followed by haematological improvement and follow‐up MRV demonstrated progressive venous sinus recanalisation, visual impairment remained with established optic atrophy. The mechanism of visual loss could not be definitively established, although ischaemic optic neuropathy was considered the most likely explanation [6–8, 13].

Long‐term management requires multidisciplinary follow‐up involving rheumatologists, neurologists, ophthalmologists and rehabilitation specialists [13]. Regular monitoring of disease activity, immunosuppressive therapy, anticoagulation and recurrent thrombotic complications is essential. Consider appropriate neuro‐ophthalmological assessment and visual rehabilitation for patients with significant residual visual impairment.

Overall, this case highlights that extensive CVST may occur in active SLE despite negative aPL testing and may present with severe neurological manifestations. Early MRI/MRV and timely treatment are essential, while anticoagulation in severe thrombocytopaenia requires careful assessment of thrombotic and bleeding risks. In this patient, treatment was followed by venous sinus recanalisation and neurological improvement, although visual recovery was limited.

## Author Contributions

Md. Gias Uddin: conceptualisation, methodology, investigation, data curation, writing–original draft and visualisation.

Mohammad Abul Kalam Azad: conceptualisation, methodology, investigation, supervision, writing–review and editing and project administration.

Md. Masudul Hassan: investigation, clinical interpretation and writing–review and editing.

Md Moinuddin Sohel: investigation, clinical interpretation and writing–review and editing.

Ismail Hossain: investigation, data curation and writing–review and editing.

Minhaj Rahim Choudhury: investigation, clinical interpretation, supervision and writing–review and editing.

All authors contributed to the conception and preparation of the case report.

## Funding

Open Access funding enabled and organized by Projekt DEAL.

## Disclosure

All authors approved the final version of the manuscript and agreed to be accountable for all aspects of the work.

## Ethics Statement

This single‐patient case report did not require ethical approval under applicable institutional policy.

## Consent

The patient provided written informed consent for publication of this case report and any accompanying clinical images.

## Conflicts of Interest

The authors declare no conflicts of interest.

## Data Availability

The clinical information supporting the findings of this case report is available from the corresponding author upon reasonable request. The data are not publicly available due to privacy and confidentiality restrictions. Relevant data may be made available to the journal and reviewers for verification or clarification, subject to appropriate ethical and confidentiality requirements.
